# The Strengths of Scanning Electron Microscopy in Deciphering SARS-CoV-2 Infectious Cycle

**DOI:** 10.3389/fmicb.2020.02014

**Published:** 2020-08-19

**Authors:** Djamal Brahim Belhaouari, Anthony Fontanini, Jean-Pierre Baudoin, Gabriel Haddad, Marion Le Bideau, Jacques Yaacoub Bou Khalil, Didier Raoult, Bernard La Scola

**Affiliations:** ^1^Microbes, Evolution, Phylogeny and Infection (MEPHI), UM63, Institut de Recherche pour le Développement (IRD), Assistance Publique – Hôpitaux de Marseille (AP-HM), Aix-Marseille University, Marseille, France; ^2^IHU Méditerranée Infection, Marseille, France

**Keywords:** SARS-CoV-2, infectious cycle, Vero E6 cells, scanning electron microscopy, Coronavirus

## Abstract

Electron microscopy is a powerful tool in the field of microbiology. It has played a key role in the rapid diagnosis of viruses in patient samples and has contributed significantly to the clarification of virus structure and function, helping to guide the public health response to emerging viral infections. In the present study, we used scanning electron microscopy (SEM) to study the infectious cycle of SARS-CoV-2 in Vero E6 cells and we controlled some key findings by classical transmission electronic microscopy (TEM). The replication cycle of the virus was followed from 1 to 36 h post-infection. Our results revealed that SARS-CoV-2 infected the cells through membrane fusion. Particles are formed in the peri-nuclear region from a budding of the endoplasmic reticulum-Golgi apparatus complex into morphogenesis matrix vesicae. New SARS-CoV-2 particles were expelled from the cells, through cell lysis or by fusion of virus containing vacuoles with the cell plasma membrane. Overall, this cycle is highly comparable to that of SARS-CoV. By providing a detailed and complete SARS-CoV-2 infectious cycle, SEM proves to be a very rapid and efficient tool compared to classical TEM.

## Introduction

The SARS-CoV-2 (COVID-19) outbreak started in late December 2019 in China and has since reached a global pandemic (Zhu et al., 2020), leading to a worldwide battle against COVID-19. SARS-CoV-2 is a novel β-coronavirus belonging to the sarbecovirus subgenus of Coronaviridae family (Schoeman and Fielding, 2019; Zhu et al., 2020). Coronaviruses are enveloped viruses with a positive sense, single-stranded RNA genome (Schoeman and Fielding, 2019). One of the first methods used for coronaviruses detection was electron microscopy (EM), which has been a reliable tool for the classification of viruses according to their ultra-structure (Hazelton and Gelderblom, 2003; Curry et al., 2006). The characteristic morphology of crown-like structures detected by EM explains the name of Coronaviridae family (Golding et al., 2016) observed as widely spaced club-shaped projections surrounding the virus envelope, thus forming a crown aspect in negative staining protocols (Almeida and Tyrrell, 1967; Oshiro et al., 1971). Coronaviruses have the largest genomes among RNA viruses, with genome sizes ranging from 26 to 32 kb in length. These viruses primarily infect birds and mammals, and can also infect humans, causing respiratory and enteric diseases, such as upper respiratory tract infections and lower respiratory tract infections (bronchitis, pneumonia, and severe acute respiratory syndrome (SARS). EM is a powerful tool in the field of microbiology, because of its resolution power as compared to light microscopy (Koster and Klumperman, 2003). EM contributed significantly to the clarification of viruses structure and function and has played a key role in the rapid diagnosis of viruses in various samples (Goldsmith and Miller, 2009). The ability of EM to detect unknown and unsuspected organisms has made it a suitable tool to guide the public health response during previous outbreaks. Transmission electron microscopy (TEM) was extensively used to describe the morphology or the morphogenesis of SARS-CoV (Ng et al., 2003; Qinfen et al., 2004), MERS-CoV (Kim et al., 2016; Park et al., 2016; Alsaad et al., 2018) or, more recently, SARS-CoV-2 (Caly et al., 2020; Colson et al., 2020; Kim et al., 2020; Zhu et al., 2020).

Scanning electron microscopy (SEM) is another powerful tool for microbiological research and diagnosis of infectious diseases (Golding et al., 2016). We already demonstrated its strengths for ultra-rapid microscope imaging of SARS-CoV-2 when pandemic first reached France (Colson et al., 2020). Here, we used SEM for its capacity to rapidly screen SARS-CoV-2-infected Vero cells in resin ultra-thin sections, allowing the ultrastructural detailed analysis of SARS-CoV-2 throughout the whole infectious cycle.

## Materials and Methods

### Cell Culture-Virus Infectious Cycle

Vero E6 cells were grown to monolayer in 25 cm^2^ culture flasks in Dulbecco’s Modified Eagle’s Medium supplemented with 10% fetal bovine serum for 2–3 days at 37°C. For the viral infection cycle, the culture medium were removed, and the cells were inoculated with SARS-CoV-2 at a multiplicity of infection (MOI) of 1. After incubation at 37°C for 45 min, the supernatant was removed. This marked time 0 (H0). For later time points, infected cells were incubated at 37°C in medium culture. Post-infection time points were: 1, 2, 3, 4, 5, 6, 12, 24, and 36 h post infection. For each time point, infected cells were detached by using 500 μl of trypsin and pelleted by centrifugation at 500 × g for 10 min.

### Scanning and Transmission Electron Microscopy

For electron microscopy infected Vero cells were fixed at least for 1 h with glutaraldehyde 2.5% in 0.1M sodium cacodylate buffer. For resin embedding, cells were washed three times with a mixture of 0.2M saccharose/0.1M sodium cacodylate. Cells were post-fixed for 1 h with 1% OsO4 diluted in 0.2M Potassium hexa-cyanoferrate (III) / 0.1M sodium cacodylate solution. After three 10 min washes with distilled water, the cells were gradually dehydrated with ethanol by successive 10 min baths in 30, 50, 70, 96, 100, and 100% ethanol. Substitution was achieved by successively placing the cells in 25, 50, and 75% Epon solutions for 15 min. Cells were placed for 1 h in 100% Epon solution and in fresh Epon 100% over-night under vacuum at room-temperature. Polymerization occured with cells in 100% fresh Epon for 72 h at 60°C. All solutions used above were 0.2 μm filtered. Ultrathin 70 nm sections were cut using a UC7 ultramicrotome (Leica) and placed on HR25 300 Mesh Copper/Rhodium grids (TAAB, United Kingdom). Sections were contrasted according to Reynolds (1963). For scanning electron microscopy (SEM), grids with sections were mounted on double-sided tape on glass slide for sequential observation of different time-points and they were platinum-coated with a MC1000 sputter coater (Hitachi) for 40 s at 10 mA. Electron micrographs were obtained on either SU5000 SEM (Hitachi High-Tech, HHT, Japan) operated between 7 and 10 kV accelerating voltage, in high-vacuum and observation mode (spot size 30), between 4.6 and 4.9 mm average working distance with BSE detector, and magnifications ranging from ×5,000 to ×100,000 or Tecnai G2 TEM (Thermo-Fischer/FEI) operated at 200 keV equipped with a 4096 × 4096 pixels resolution Eagle camera (FEI).

## Results

### SARS-CoV-2 Cell Entry

**FIGURE 1:**
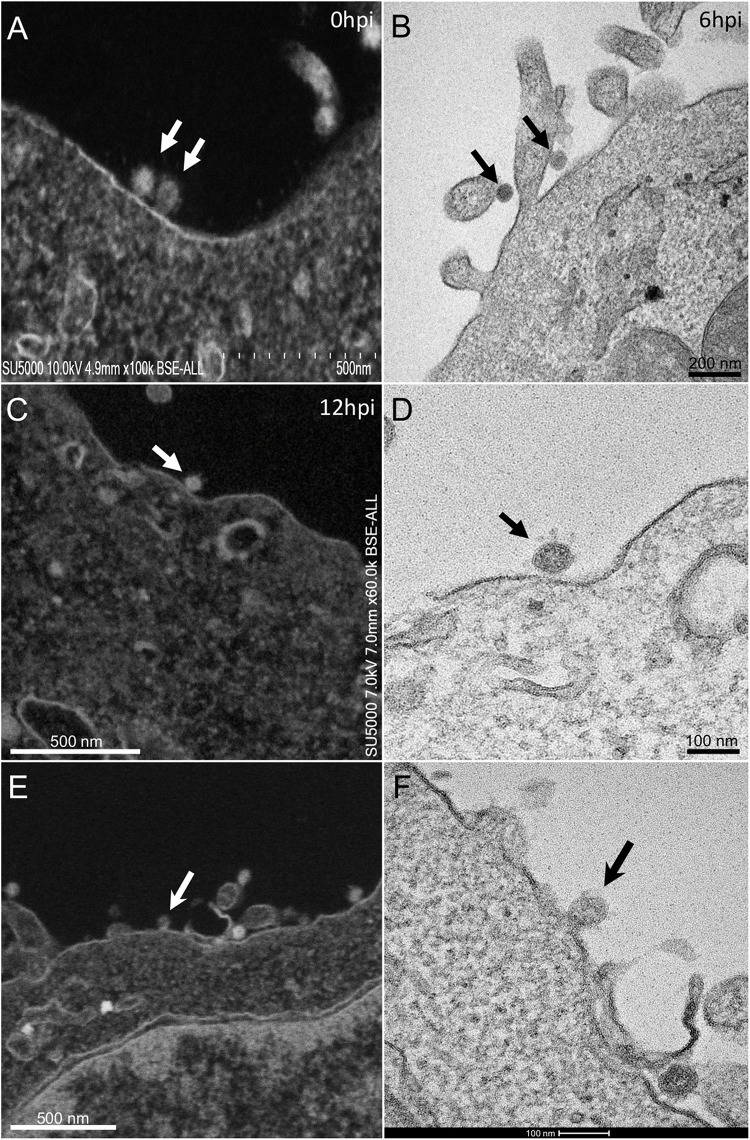
SARS-CoV-2 infected Vero E6 cells At early post-infection time-point with virus **(A,B)** at the periphery of Vero E6 cells (arrows). **(C,D)** SEM **(C)** and TEM **(D)** views of the same cellular region with a SARS-CoV-2 particle (arrow) attached to the plasma membrane, the corona spikes of which are located between the particle and the cell plasma membrane. **(E,F)** SEM **(E)** and TEM **(F)** views of the same cellular region showing SARS-CoV-2 virus particles attached to the cell plasma membrane; one particle (arrow in **E,F**) is glued to the plasma membrane, fusing with the cell plasma membrane.

**FIGURE 2:**
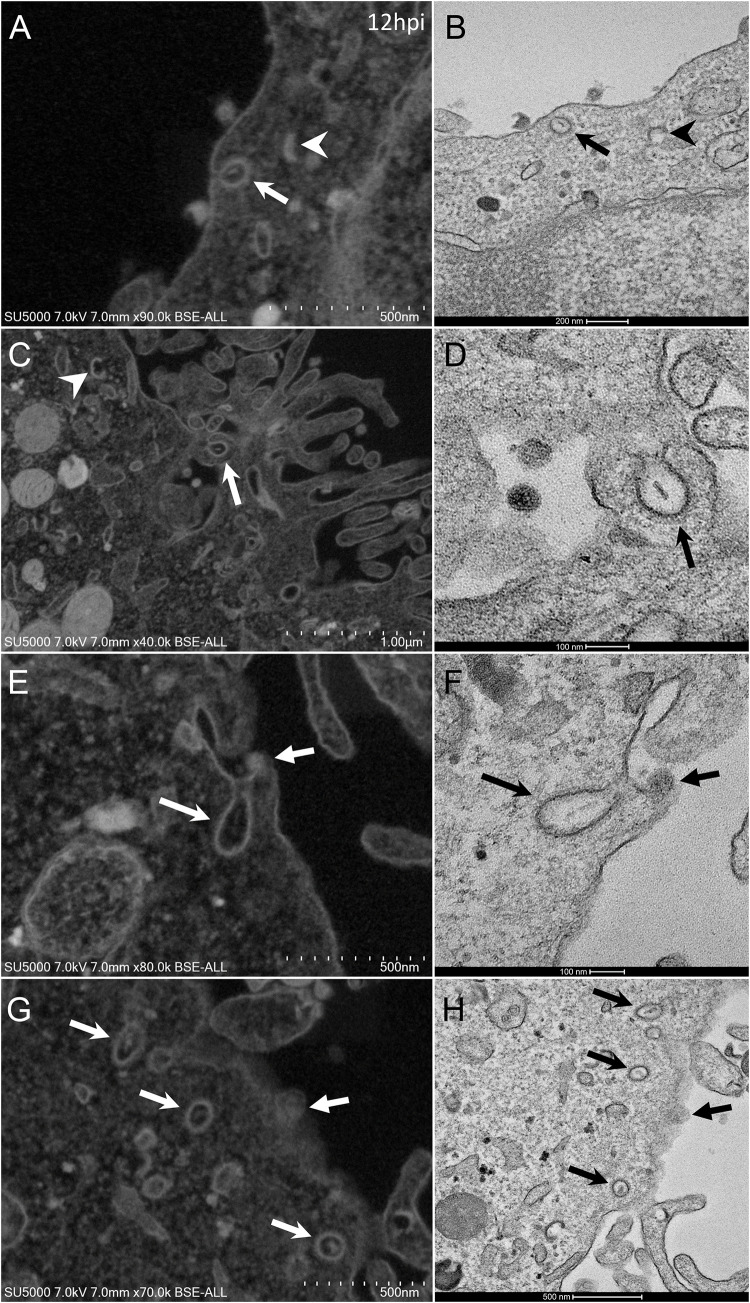
SARS-CoV-2 infected Vero E6 cells with endocytic vesicles in the cytoplasm. **(A–F)** SEM **(A,C,E,G)** and TEM **(B,D,F,H)** views of the same cellular regions with clathrin-coated vesicles (arrows) containing rod-like amorphous material. Crescent-like electron-dense structures (arrowhead in **A**) were often depicted in infected cells cytoplasm. Solid arrows **(E–H)** point to glued SARS-CoV-2 particles on cells plasma membrane.

At early post-infection time-point SARS-CoV-2 virions were detected by SEM and TEM, located at the surface of the cells (Figure 1). In those cells, we did not notice viral morphogenesis features, and particles were seen i) attached, with their corona spikes located between the particle and the plasma membrane (Figures 1C,D) or ii) less electron-dense, with the envelope fusing with the plasma membrane (Figures 1E,F). Endocytic vesicles with typical clathrin-coated pits were often observed below particles attached to the plasma membrane (Figures 2E,F). In these forming endocytic particles, rod-like amorphous material was present (Figure 2). This kind of material was also observed in clathrin-coated endocytic vesicles located more deeply in the cell cytoplasm (Figures 2G,H). We also observed in the cytoplasm of cells with particles at the plasma membrane electron-dense crescent-shaped intracellular structures (Figures 2A–C), that were also present in control, non-infected cells (Supplementary Figure 1). A very few virus-like particles were also observed inside the cells, in endoplasmic reticulum (ER)-derived peripheral canaliculi (not shown). From H1 to H5 SARS-CoV-2 virions were not detected in the ultra-thin sections.

**FIGURE 3:**
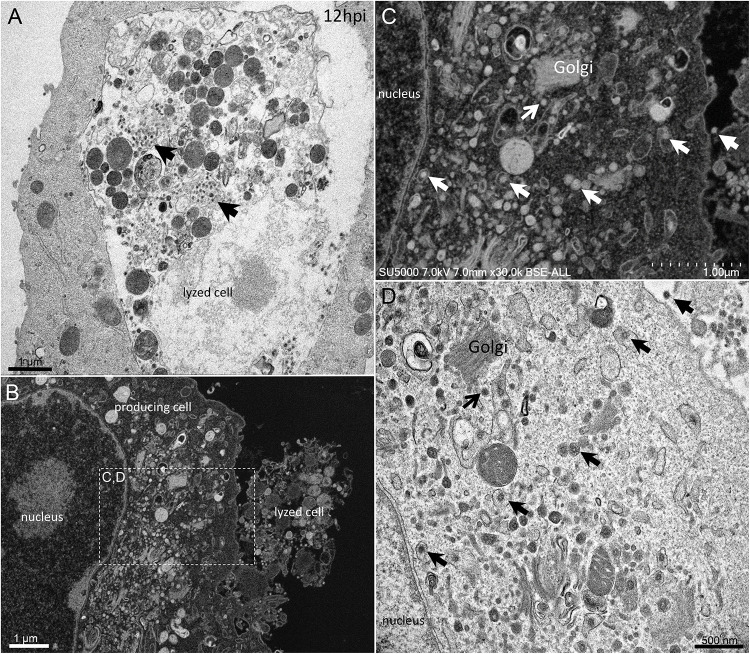
SARS-CoV-2 infected Vero E6 cells at 12 h post-infection. **(A)** Cell in lytic process with vacuole filled with nascent particles (arrows). **(B)** producing-virus cell adjacent to lysed cell. **(C,D)** zooming on virus-producing cell shows mature SARS-CoV-2 particles with their corona spikes (arrows) in small cytoplasmic vacuoles between the nucleus and the cell periphery, observed by SEM **(C)** or TEM **(D)** in a cell. Extracellular particles attached to the plasma membrane (arrows).

From H12, SARS-CoV-2 virions were found attached to lyzed cells containing vacuoles filled with nascent particles (Figure 3A) or attached to cells containing mature SARS-CoV-2 particles with corona spikes located in small cytoplasmic vacuoles, between the nucleus and the cell periphery (Figures 3B–D) and also attached to cells without morphogenesis features (Figures 1C,F). From H18 onwards, most of the viruses attached to cell plasma membranes were seen in virus-producing-cells, which were lyzed or intact.

### SARS-CoV-2 Morphogenesis

**FIGURE 4:**
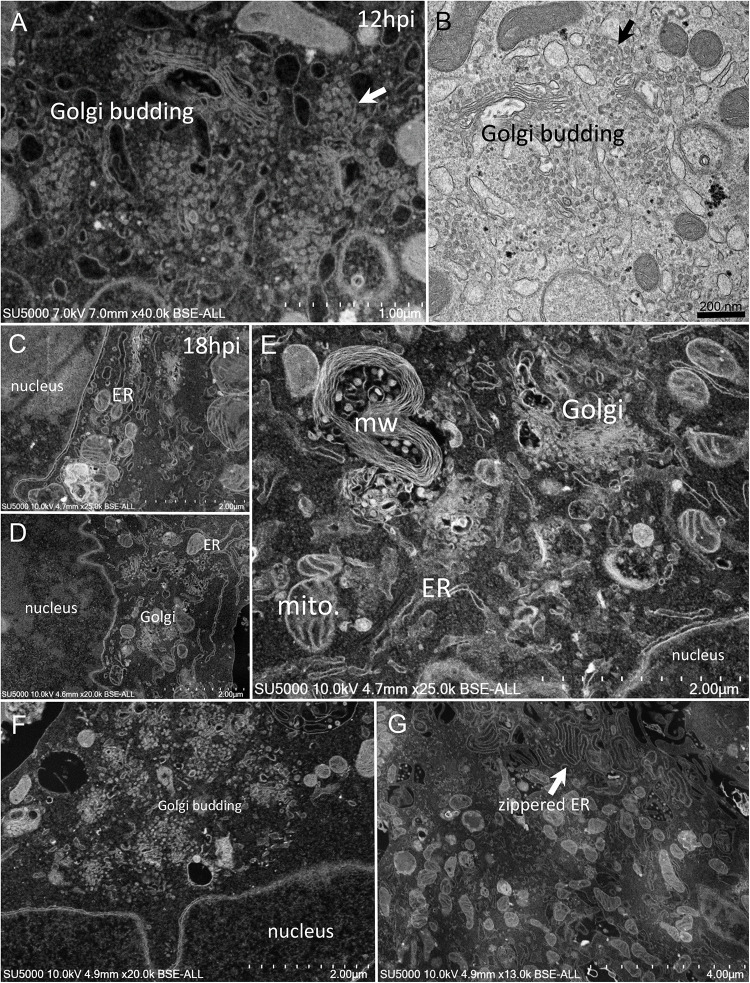
SARS-CoV-2 infected Vero E6 cells at 12–18 h post-infection. **(A,B)** SEM **(A)** and TEM **(B)** views of the same cell region showing extreme Golgi apparatus budding as well as a vacuole filled with nascent particles close to the Golgi apparatus. **(C,D)** Thick and distorted endoplasmic reticulum (ER) tubules were observed by SEM at peri-nuclear location and at the cells periphery. **(E)** SEM view of the extensive enlargement and budding of the ER and Golgi apparatus, as well as a myelin-like membranes whorl (mw) close to the Golgi apparatus and mitochondria (mito.) in the perinuclear region. **(F)** SEM low-magnification view of Golgi budding between a nucleus and the peripheral plasma membrane. **(G)** SEM image of zippered endoplasmic reticulum (ER) at apical location (arrow).

First, swollen nuclear membrane, endoplasmic reticulum (ER) and Golgi apparatus (GA) organelles were the most striking features of SARS-CoV-2 infected cells (Figure 4). Thick and distorted ER tubules were observed at peri-nuclear locations between the nucleus and the GA (Figures 4C,D), and also at peripheral locations below the plasma membrane, where the ER could be seen as zippered (Figure 4G). When intact, the GA was found at peri-nuclear locations, with Golgi stacks lying parallel to the ER and the nuclear membranes (Figures 4C,D). As infection progressed, the GA was found budding between large ER tubules, resulting in multiple Golgi-derived nascent particles and a loss of intact GA stacks (Figures 4A,B,F). The extent of GA budding was variable from a cell to another, being generally proportional to its distance from the nucleus. At early infection stages, myelin-like membranes whorls were present at proximity of the Golgi apparatus (Figure 4E). These whorl types are probably not a typical feature of infected cells, as they were also observed in uninfected cells (Supplementary Figure 1). We noticed abundant mitochondria in apical regions of both non-infected and infected cells at all stages, around the ER and Golgi-rich regions (not shown).

**FIGURE 5:**
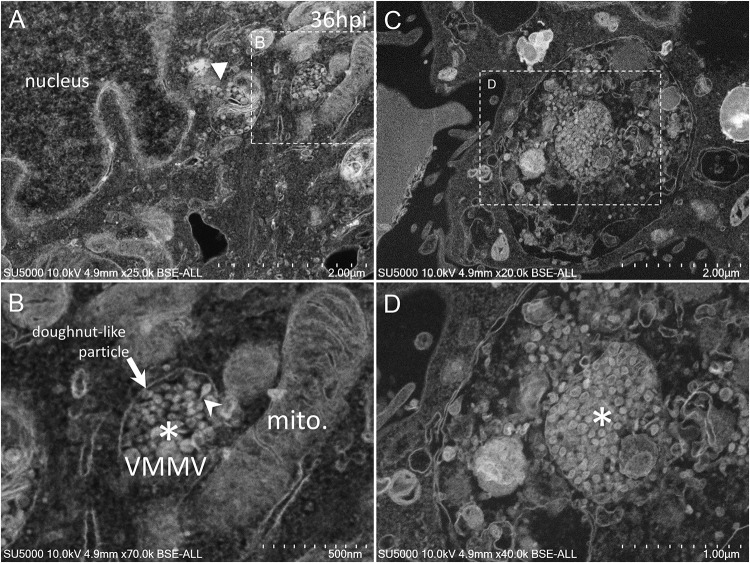
SARS-CoV-2 infected Vero E6 cells at 36 h post-infection. **(A,B)** SEM views of doughnut-like particles (arrow) with a pronounced electron-opaque edge observed at peri-nuclear locations dispersed into the cytoplasm (solid arrowhead), or within vacuoles, which seemed derived from the ER, forming virus morphogenesis matrix vesicae (VMMV). VMMV were observed as opened sacs (asterisk in **B**), assembling next to the nucleus **(A,B)**. Protrusions of the doughnut-like particles could be observed in the VMMV (arrowhead in **B**). **(C,D)** VMMV observed as a closed sac (asterisk in **D**) in a larger vacuole in the cytoplasm. **(A–D)** SEM images.

Golgi-derived doughnut-like particles with a pronounced electron-opaque edge were observed at peri-nuclear locations (Figures 5A,B), dispersed into the cytoplasm, as well as entering vacuoles, which seemed to be derived from the ER (Figures 5A,B). Such forming virus morphogenesis matrix vesicae (VMMV) (Qinfen et al., 2004), filled with doughnut-like particles, were observed as open sacs, assembling next to the nucleus (Figures 5A,B), or closed sacs adjacent to or distant from the nucleus, in the cytoplasm or in the vacuoles (Figures 5C,D). Nascent particles were first observed at H12 in only a few cells (Figure 3), to a lesser extent than in more advanced times of infection. Doughnut-like particles were 70 ± 6 nm in diameter (*n* = 100), devoid of corona spikes. Their shape was not perfectly round when observed in the assembling opened sacs, and these particles could present filopodia-like protrusion (Figure 5B).

**FIGURE 6:**
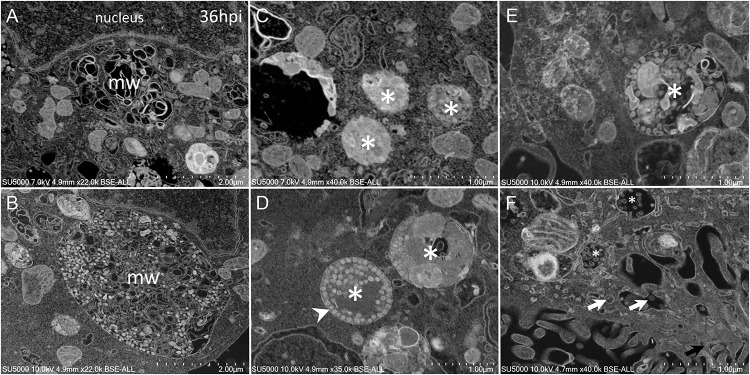
SARS-CoV-2 infected Vero cells at 36 h post-infection **(A)** SEM depiction of an extensive peri-nuclear membrane whorls (mw) network, with larger inner-membranous distances as infection progresses. **(B)** Nascent virions particles (≈70 nm) observed by SEM mixed with membranes whorls in large bags. **(C–F)** SARS-CoV-2-morphogenesis matrix vesicae (asterisk) with different appearances, with nascent particles (≈70 nm) located in more or less large vacuoles, containing more or less electron-dense materials (asterisk). **(F)** Translucent VMMV/vacuoles with well-individualized virions particles (white arrows).

As the infection progressed, we observed an extensive network of membrane whorls, with large inter-membranous distances when compared to previous “small” membrane whorls (Figure 6A). These intermingled membranes were lying at the level of concave nuclear indentations. Nascent virions could be found mixed with such membranes whorls in large bags (Figure 6B). The appearance of the VMMV was variable, with nascent particles located in more or less large vacuoles, containing more or less electron-dense material (Figures 6C–F). The electron-density of these vacuoles filled with nascent particles was correlated with the heterogeneity of these vacuoles: electron-dense virions-filled vacuoles were homogenous (Figures 6C,D), while electron-lucent vacuoles contained virions particles as well as heterogeneous materials such as membranes, or distorted compartments (Figures 6E,F). VMMV located below the plasma membrane were frequently seen translucent, with well-individualized virions particles (Figure 6F). Particles could arrange as circular chains lying on the internal surface of the VMMV (Figure 6D).

We also noticed at nucleus margins of infected cells, round and empty objects with ± a punctate pattern at their center (Supplementary Figures 2A–F). These objects 100–120 nm in diameter were present at locations where the nuclear membrane was not clearly delineated, in contrast to adjacent regions where the nucleus double membrane was properly seen. These features could be observed in transverse (Supplementary Figures 2A–E,G) or in tangential sections of the nuclei (Supplementary Figures 2F,H), the objects being in the latter case, located in an electron-dense chromatin-like material, which we called nuclear matrix. These objects were also observed, to a lesser extent, in uninfected cells (Supplementary Figure 3).

### SARS-CoV-2 Cell Exit

**FIGURE 7:**
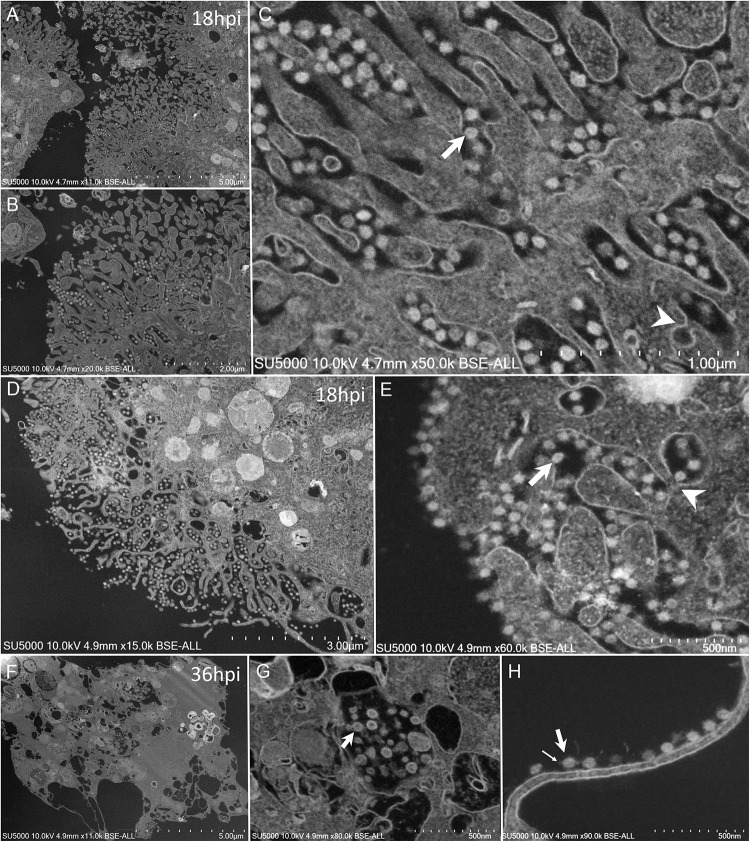
SEM of SARS-CoV-2 mature particles at 18–36 h post-infection. observed at intra- and extra- cellular locations. **(A–C)** Viral mature particles lying between cellular microvilli. **(D,E)** Virus particles within smooth vesicles at the cell periphery and eventually fused with the cell membrane (arrowhead) to release the virus (arrow). **(F,G)** Viral particles in translucent vacuoles or attached to the plasma membrane of a lysed cell. Small arrow points to a SARS-CoV-2 corona spike.

Mature SARS-CoV-2 particles 80 ± 7 nm in diameter (*n* = 100) were observed as spiky round to hexagonal electron-dense particles (Figure 7). Mature SARS-CoV-2 particles were observed at extra- and intra-cellular locations: in translucent VMMV or vacuoles of early-infected intact- cells (Figure 6F), and later found lying between cellular microvilli (Figures 7A–C), in vacuoles (Figure 7G), as well as on the surface of lysed cells (Figures 7F,H). Intracellular compartments filled with mature virions were observed channeling with the apical side of the Vero cells at the base of the microvilli (Figures 7C,E).

## Discussion

Our results show spiky round to hexagonal 80 nm in diameter electron-dense mature SARS-CoV-2 particles, similar to the previously described SARS-CoV-2 virions (Colson et al., 2020; Kim et al., 2020; Zhu et al., 2020). Previous analysis of ultrathin sections by TEM of SARS-CoV-2 infected- cells showed virus particles in inclusion bodies in human airway cells (Zhu et al., 2020), as well as in a wide range of intracellular organelles, especially in vesicles of Vero cells (Caly et al., 2020; Kim et al., 2020). Here, thanks to our SEM analysis, we were able to show the similarity between SARS-CoV-2 and SARS-CoV infectious cycles, with some exceptions.

In our experiments, At early post-infection time-point, SARS-CoV-2 virions were seen attached at cells plasma membrane. The absence of SARS-CoV-2 virions observation from H1 to H5 is probably related to the very brief binding of infectious particles to the cell surface, their rapid reduction once attached to the cells and a consecutive eclipse phase. From H12 post-infection, different cell profiles were seen: no producing-virus cells, virus-producing cells and lysed cells. This variability in the stage of infection is probably due to a desynchronized virus infection due to cells not being infected at the same time or to a low infectious titer of virus used to infect the cells. At H12, attached particles to cell plasma membranes were seen in non-virus-producing cells, probably corresponding to cells being infected by neo-synthesized virions produced by adjacent infected cells, and also in virus-producing cells with morphogenesis features. For this latter case, it is not known if virions attachment onto already infected cells would yield further productive cycles of replication.

Regarding SARS-CoV-2 cell entry process, particles were observed attached at cells plasma membrane, located at cells apical sides, similarly to SARS-CoV (Rossen et al., 1994). Full SARS-CoV-2 particle endocytosis was not observed, which is consistent with previous studies conducted on SARS-CoV particles infecting cells by membrane fusion (Oshiro et al., 1971; Ng et al., 2003; Qinfen et al., 2004). In fact, we observed a possible fusion of SARS-CoV-2 particles with the cells plasma membranes. Our images suggest that these attached particles were probably caught transferring their content inside the cell cytoplasm. The role of the clathrin endocytic vesicles containing amorphous material, as intermediate receptacles of SARS-CoV-2 genomic content after fusion of the particles with cells plasma membranes, is likely to be part of the SARS-CoV-2 nucleocapsid cell entry process.

Regarding SARS-CoV-2 morphogenesis, previous studies reported nuclear localization of SARS-CoV proteins or particles (Zhang et al., 2003; Qinfen et al., 2004; Yuan et al., 2005). Here, we did not observe SARS-CoV-2 particles inside the nucleus. We observed round objects in a nuclear matrix without distinctive membranous limits, in infected as well as in non-infected cells (Supplementary Figures 2, 3). These objects likely correspond to nuclear pore complexes according to morphology (round ± punctate pattern) and diameter (Bardina et al., 2009; Lin and Hoelz, 2019). The abundance of mitochondria next to the ER and GA budding regions, where SARS-CoV-2 morphogenesis occurred, could provide energy for viral multiplication (de Castro et al., 2013). It was assumed that doughnut-shaped electron-dense structures (also observed in SARS-CoV infectious cycle studies) probably correspond to assemblies of virus genomes together with helical nucleocapsids (Ng et al., 2003). As in SARS-CoV-2 infected cells, myelin-like membrane whorls have been previously described in SARS-CoV infected cells, closely associated with nascent particles (Ng et al., 2003). Although these membrane whorls were also present in uninfected cells, we hypothesize that these compartments may be derived from the ER and/or may be part of an auto-phago-(lyso) somal process, both scenarios providing a support for virions packaging and trafficking until further extracellular release. The electron-density and homogeneity difference of the VMMV containing viral particles may be related to the pH of these compartments and/or to the maturation level of the virions. The circular aspect of SARS-CoV-2 assemblies inside some VMMVs may reflect the presence of mature particles, compared to immature and dispersed particles in less organized VMMV. As for SARS-CoV infected cells, one of the most obvious ultrastructural changes in SARS-CoV-2 infected cells was the proliferation of the Golgi complexes and related vesicles, accompanied by swelling of some of the Golgi sacs. We also found that VMMVs are most probably derived from the ER. It was shown that SARS-CoV nucleocapsids assemble in the ER and mature by budding into smooth vesicles derived from the GA. In parallel, the GA swells to form smooth vesicles that incorporate the VMMV along with their nucleocapsids (Oshiro et al., 1971; Patterson and Macnaughton, 1982; Zhang et al., 2003; Qinfen et al., 2004; Siu et al., 2008). Rather, our images suggest that nascent particles may bud in the cytoplasm, followed or simultaneous to the filling of the ER-derived VMMV by immature Golgi-derived virions. Nevertheless, the involvement of the Endoplasmic Reticulum – Golgi Apparatus complex witnessed here, especially the extreme budding of the Golgi apparatus, into the morphogenesis of the SARS-CoV-2, is consistent with what was demonstrated in chloroquine’s efficacy against SARS-CoV-2 *in vitro* (Andreani et al., 2020; Wang et al., 2020). Indeed, chloroquine is a weak base which interferes with cell trafficking by increasing the pH of intracellular compartments (Devaux et al., 2020), especially lysosomes, and was shown to severely affects the endo-lysosomal system and the Golgi complex *in vitro* and *in vivo* (Mauthe et al., 2018).

Regarding SARS-CoV-2 cell exit, mature virions exited the cells at their apical sides, as observed for SARS-CoV (Rossen et al., 1994). The release of mature particles occurred passively in lysed cells or by fusion of the internal compartments with the plasma membrane in intact cells, as previously described (Oshiro et al., 1971; Ng et al., 2003; Qinfen et al., 2004).

Scanning electron microscopy (SEM) has shown here two main advantages over transmission electron microscopy (TEM) for studying SARS-CoV-2 life cycle. The first advantage came from the possibility to load at one time in the microscope several grids with ultra-thin sections of resin-embedded infected cells, corresponding to different post-infection times. All post infection times were thus accessible within 10 min in the SEM. The second advantage was that after SEM electron beam alignment and focus were adjusted, SEM screening of the ultra-thin sections was fast, compared to TEM, as only minor adjustments of the focus distance were needed when zooming on cells of interest or when moving from one grid to another. For TEM, focus adjustment was generally required for each position and magnification, and thus more time consuming. The whole SARS-CoV-2 infectious cycle was thus accessible at once, in a few hours of observation (4.5 h for 8 time-points, acquiring 320 micrographs), with possibilities to image at the cell population, cellular and sub-cellular levels. A similar screening of the same grids by TEM was more time consuming (around 8 h).

## Conclusion

In conclusion, SEM has proven to be a rapid and effective tool for studying the SARS-CoV-2 infectious cycle in Vero cells. Further studies employing the same straightforward methodology may help understand at the cellular level the impact of pharmacological reagents on SARS-CoV-2 life cycle to better control this global pandemic.

## Data Availability Statement

The raw data supporting the conclusions of this article will be made available by the authors, without undue reservation, to any qualified researcher.

## Author Contributions

DB, J-PB, GH, ML, AF, and JB did the experiments and analyzed the data. DB, J-PB, and GH wrote the manuscript. J-PB, JB, DR, and BL conceived the project, supervised the experiments, and wrote the manuscript. All authors contributed to the article and approved the submitted version.

## Conflict of Interest

DR was a consultant for the Hitachi High-Tech Corporation. The remaining authors declare that the research was conducted in the absence of any commercial or financial relationships that could be construed as a potential conflict of interest.

## Supplementary Material

FIGURE S1 SEM of uninfected Vero E6 cell showing electron-dense crescent-shaped in the cytoplasm (solid arrowheads), and a myelin-like membranes whorl (mw) in the perinuclear region.

FIGURE S2 SARS-CoV-2 infected Vero E6 cells at 12–24 h post-infection. **(A–H)** SEM **(A,G,H)** and TEM **(B–F)** images showing numerous round and empty objects (arrows) at nucleus margins (n.m). **(A–E,G)** Images of nuclear membrane budding sites observed in transverse views. **(F,H)** Images of nuclear membrane budding sites in tangential views. SARS-CoV-2 particles can be seen outside the cells at cell plasma membranes [solid arrows in **(A,B)**, which correspond to the same cellular region].

FIGURE S3 SEM of uninfected Vero E6 cell image showing round and empty objects (arrows) at nucleus margins **(A,B)** (arrows).
