# Supplementary figures and images for: The Strengths of Scanning Electron Microscopy in Deciphering SARS-CoV-2 Infectious Cycle
Source: Front Microbiol. 2020 Aug 19;11:2014. doi: 10.3389/fmicb.2020.02014 (PMC7466455; doi:10.3389/fmicb.2020.02014)

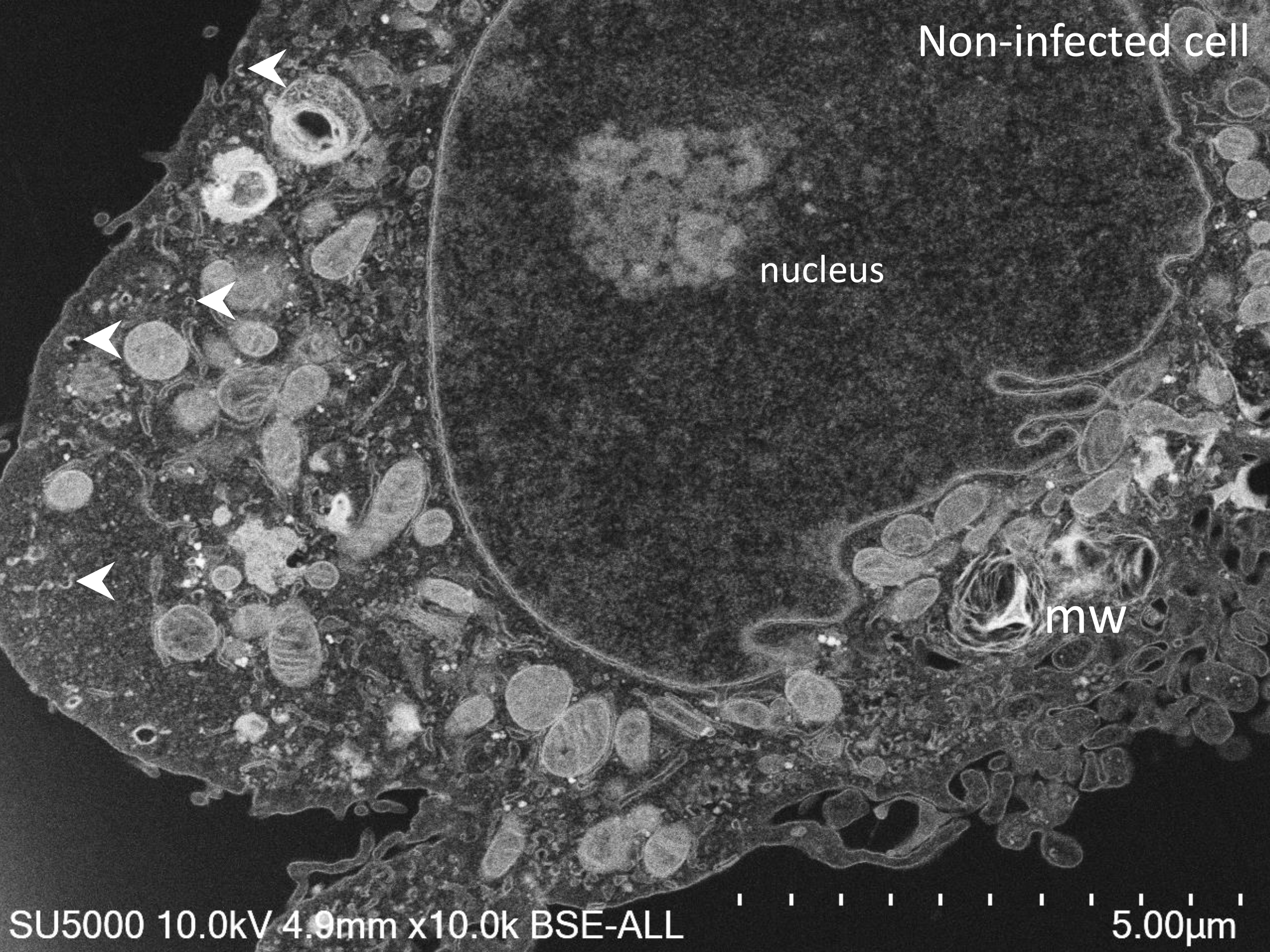

Supplement: FIGURE S1 — SEM of uninfected Vero E6 cell showing electron-dense crescent-shaped in the cytoplasm (solid arrowheads), and a myelin-like membranes whorl (mw) in the perinuclear region. [file Image_1.TIF]

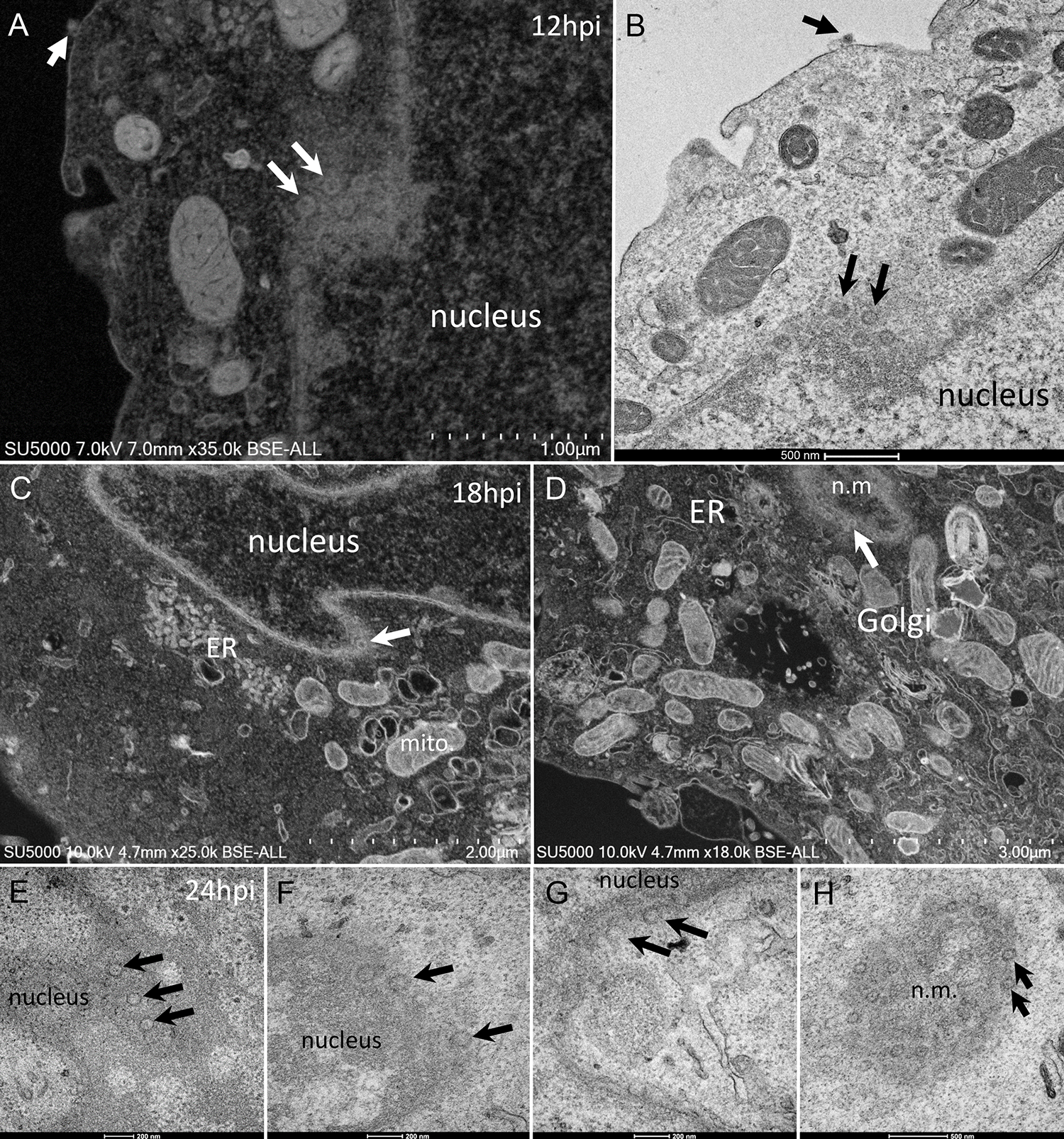

Supplement: FIGURE S2 — SARS-CoV-2 infected Vero E6 cells at 12–24 h post-infection. (A–H) SEM (A,G,H) and TEM (B–F) images showing numerous round and empty objects (arrows) at nucleus margins (n.m). (A–E,G) Images of nuclear membrane budding sites observed in transverse views. (F,H) Images of nuclear membrane budding sites in tangential views. SARS-CoV-2 particles can be seen outside the cells at cell plasma membranes [solid arrows in (A,B), which correspond to the same cellular region]. [file Image_2.TIF]

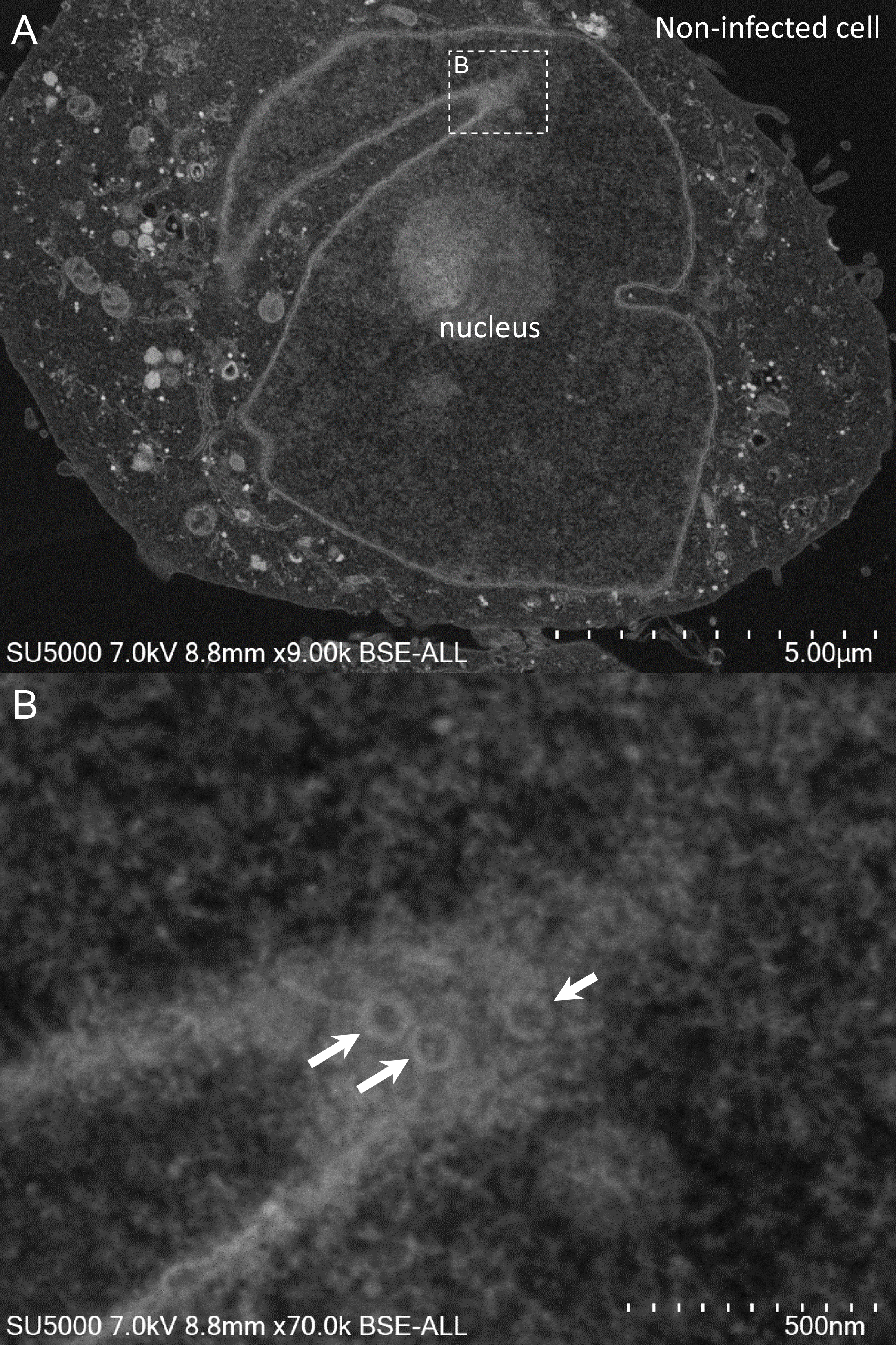

Supplement: FIGURE S3 — SEM of uninfected Vero E6 cell image showing round and empty objects (arrows) at nucleus margins (A,B) (arrows). [file Image_3.TIF]
